# Recorded Mental Health Recovery Narratives as a Resource for People Affected by Mental Health Problems: Development of the Narrative Experiences Online (NEON) Intervention

**DOI:** 10.2196/24417

**Published:** 2021-05-27

**Authors:** Mike Slade, Stefan Rennick-Egglestone, Joy Llewellyn-Beardsley, Caroline Yeo, James Roe, Sylvia Bailey, Roger Andrew Smith, Susie Booth, Julian Harrison, Adaresh Bhogal, Patricia Penas Morán, Ada Hui, Dania Quadri, Clare Robinson, Melanie Smuk, Marianne Farkas, Larry Davidson, Lian van der Krieke, Emily Slade, Carmel Bond, Joe Nicholson, Andrew Grundy, Ashleigh Charles, Laurie Hare-Duke, Kristian Pollock, Fiona Ng

**Affiliations:** 1 School of Health Sciences Institute of Mental Health University of Nottingham Nottingham United Kingdom; 2 National Institute for Health Research ARC East Midlands University of Nottingham Nottingham United Kingdom; 3 NEON Lived Experience Advisory Panel Nottingham United Kingdom; 4 Department of Personality, Assessment and Psychological Treatment University of Deusto Bilbao Spain; 5 GKT School of Medical Education King’s College London London United Kingdom; 6 Centre for Primary Care & Public Health Pragmatic Clinical Trials Unit Queen Mary University of London London United Kingdom; 7 Department of Medical Statistics London School of Hygiene and Tropical Medicine London United Kingdom; 8 College of Health and Rehabilitation Sciences Boston University Boston, MA United States; 9 Yale School of Medicine Yale University New Haven, CT United States; 10 University Medical Center Groningen University Center of Psychiatry University of Groningen Groningen Netherlands; 11 Department of Computer Science University of Oxford Oxford United Kingdom; 12 Nottingham University Business School University of Nottingham Nottingham United Kingdom; 13 School of Humanities University of Nottingham Nottingham United Kingdom; 14 School of Health Sciences University of Nottingham Nottingham United Kingdom

**Keywords:** narratives, storytelling, intervention development, mental health, online intervention, patient involvement, narrative medicine, internet, recovery, mobile phone

## Abstract

**Background:**

The internet enables sharing of narratives about health concerns on a substantial scale, and some digital health narratives have been integrated into digital health interventions. Narratives describing *recovery* from health problems are a focus of research, including those presented in *recorded* (eg, invariant) form. No clinical trial has been conducted on a web-based intervention providing access to a collection of Recorded Recovery Narratives (RRNs).

**Objective:**

This study presents knowledge produced through the development of the Narrative Experiences Online (NEON) Intervention, a web-based intervention incorporating the algorithmic recommendation of RRNs.

**Methods:**

Knowledge was gathered through knowledge integration (KI) activities. KI1 synthesized previous studies to produce the NEON Impact Model describing how accessing RRNs produces health-related outcomes. KI2 developed curation principles for the NEON Collection of RRNs through consultation with the NEON Lived Experience Advisory Panel and the curation of a preliminary collection. KI3 identified harm minimization strategies for the NEON Intervention through consultation with the NEON International Advisory Board and Lived Experience Advisory Panel. The NEON Intervention was finalized through 2 research studies (RS). In RS1, mental health service users (N=40) rated the immediate impact of randomly presented narratives to validate narrative feedback questions used to inform the recommendation algorithm. In RS2, mental health service users (n=25) were interviewed about their immediate response to a prototype of the NEON Intervention and trial procedures and then were interviewed again after 1 month of use. The usability and acceptability of the prototype and trial procedures were evaluated and refinements were made.

**Results:**

KI1 produced the NEON Impact Model, which identifies moderators (recipient and context), mechanisms of connection (reflection, comparison, learning, and empathy), processes (identification of change from narrative structure or content and internalization of observed change), and outcomes (helpful and unhelpful). KI2 identified 22 curation principles, including a mission to build a large, heterogeneous collection to maximize opportunities for connection. KI3 identified seven harm minimization strategies, including content warnings, proactive and reactive blocking of narratives, and providing resources for the self-management of emotional distress. RS1 found variation in the impact of narratives on different participants, indicating that participant-level feedback on individual narratives is needed to inform a recommender system. The order of presentation did not predict narrative feedback. RS2 identified amendments to web-based trial procedures and the NEON Intervention. Participants accessed some narratives multiple times, use reduced over the 4-week period, and narrative feedback was provided for 31.8% (105/330) of narrative accesses.

**Conclusions:**

RRNs can be integrated into web-based interventions. Evaluating the NEON Intervention in a clinical trial is feasible. The mixed methods design for developing the NEON Intervention can guide its extension to other clinical populations, the design of other web-based mental health interventions, and the development of narrative-based interventions in mental health.

## Introduction

### Background

The growing use of social media platforms has enabled the sharing of digital narratives about health and health problems on a substantial scale [1]. The sharing of digital health narratives has been examined across a range of domains, including cancer [2], chronic pain [3], and mental health [4]. Digital health narratives have also been incorporated into complex health interventions, where they can be used to structure therapeutic discussions [5], support engagement with an intervention [6], address health inequalities by giving voice to underrepresented populations [7], and reach nonmajority cultural groups or hard-to-reach populations [6-8]. Access to digital health narratives might also normalize experiences of health conditions and facilitate attitudinal change in health care staff when used as part of clinical education programs [9]. Although some interventions have integrated bespoke digital narratives curated by intervention development teams [10], others draw on the vast range of available public narratives, sometimes using algorithms to select those narratives that might provide the most therapeutic benefit for a recipient [11].

One specific focus of research has been on the subset of health narratives that describe *recovery* from health problems [12,13]. The definitions of recovery narratives vary across health domains. A systematic review of mental health defined a recovery narrative as a first-person lived experience account, which refers to events or actions over a period and which includes elements of adversity or struggle as well as self-defined strengths, successes, or survival [13]. Narratives matching this definition are henceforth referred to as recovery narratives.

Recovery narratives can be shared as part of synchronous interactions between people, and sharing a recovery narrative is a core component of the work of peer specialists [14], an established effective intervention [15]. Recovery narratives can also be shared asynchronously in invariant forms such as text (eg, prose or poetry), audio, video, and other media, including visual artwork [16]. Narratives shared asynchronously are referred to as recorded recovery narratives in the remainder of this paper. Early examples of recorded recovery narratives can be found in the 1957 book *The Plea for the Silent* [17], which was published with the intent of addressing stigma about mental health problems. Recorded recovery narratives have been shared by health service units through booklets collating stories produced by their clients [18] and in printed autobiographies [19]. When shared on the web, recovery narratives can be presented individually [20-23] or in curated collections [24]. Recovery narratives have been incorporated into collections produced by antistigma campaigns such as Time to Change in the United Kingdom [25] and into websites intended to support the recovery process, such as Here to Help in Canada [26].

Sharing a recovery narrative can provide substantial benefits for the narrator [27], and developing new stories about one’s experiences is central to the work of the hearing voices groups [28] and supports posttraumatic growth [29]. Qualitative research suggests that the elements of narratives describing recovery can also provide specific benefits to recipients, such as reducing isolation or providing hope for the future [30]. Although randomized controlled trials have been conducted on interventions incorporating digital health narratives in relation to weight loss [11] and cancer [31], no randomized controlled trial has been conducted on the use of recorded mental health recovery narratives to benefit recipients. The best interventional evidence to date is derived from a study that investigated the use of a bespoke website presenting recovery narratives, among other mental health material. This identified benefits in three domains: being inspired, knowing I am not alone, and believing recovery is possible [10]. Narratives could be received in private, but meetings with support workers were available.

### NEON Program

#### Overview

This paper describes the development of a web-based intervention that presents recorded recovery narratives. Research has been conducted as a part of Narrative Experiences Online (NEON), a 5-year program of work funded through the Programme Grants for Applied Research scheme of the National Institute of Health Research from 2017 to 2022. The aim of NEON is to investigate whether accessing recorded recovery narratives can improve health-related quality of life for people affected by mental health problems [32]. The NEON program comprises three stages: theory studies, which are completed and summarized below; intervention development studies, which are reported in this paper and integrate knowledge produced by theory studies; and 3 randomized controlled trials to evaluate the intervention. The primary outcome measure for all 3 trials was the Manchester Short Assessment of Quality of Life [33].

#### NEON Theory Studies

A systematic review developed an empirically supported definition of a mental health recovery narrative [13]. The review also used a narrative synthesis of 45 included publications to develop a conceptual framework describing the characteristics of mental health recovery narratives, grouped into nine superordinate categories: genre, positioning, emotional tone, relationship with recovery, trajectory, use of turning points, narrative sequence, protagonists, and use of metaphors. This framework was validated and extended through the analysis of 77 recovery narrative interviews [34] to provide a finalized Recovery Narrative Conceptual Framework.

A systematic review was then conducted to develop a Narrative Impact Conceptual Framework, describing the forms of impact from accessing mental health recovery narratives [1]. This review included 5 articles. Narrative synthesis was conducted to identify five transdiagnostic benefits to recipients: connectedness, a better understanding of recovery, a reduction in stigmatic views (including self-stigma), the validation of difficult personal experiences, and potentially beneficial behavioral responses, such as the initiation of more meaningful interactions with support workers. Strong affective responses can be produced in recipients of recovery narratives. The review identified one harmful outcome: emulating harmful behaviors encountered in eating disorder recovery narratives. It identified that strategies are needed to support the processing of affective responses and to minimize harmful impact. The review concluded that interventions incorporating recorded recovery narratives might be particularly relevant in areas with low population density, that is, where access to both mental health care and peers with experience of similar mental health problems may otherwise be difficult [35].

The Narrative Impact Conceptual Framework was then extended by a study that developed a long-term narrative impact model characterizing the longer-term impact of live and recorded mental health recovery narratives [36]. This study involved an iterative thematic analysis of 77 interviews in which participants told their own recovery narrative and talked about the impact of recovery narratives of others on them. Helpful changes were identified as perceptions of connectedness to the narrative or narrator (as the strongest mechanism), validation, hope, empowerment and appreciation, a reduction in stigma and self-stigma, and the initiation of a particular form of turning point [37] identified as a reference shift, where accessing narrative content leads to a rapid and radical change in how recipients view what is possible for them. Harmful changes were identified as perceptions of inadequacy (eg, if a narrative describes a recovery that the recipient thinks to be impossible), disconnection (eg, from narrators who appear to have experienced less distress than the recipient), pessimism (eg, how much recovery is possible for the recipient), and emotional burden caused by empathy with the parts of a recovery narrative that describes adversity or struggle. The model also identified factors that might moderate the impact of recovery narratives: the recipient is experiencing a crisis and the recipient perceives the recovery narrative as authentic or inauthentic. The long-term narrative impact model had two main findings. First, a universally helpful recovery narrative is unlikely to exist, as components of recovery narratives that create benefits for some, such as observing narrator achievements, can cause harm for others. Second, a careful selection of narratives for use in an intervention is not by itself a sufficient harm management strategy, and other approaches are also needed to minimize harm.

A short-term narrative impact model was then developed through an experimental study on the immediate effect of accessing recorded recovery narratives [38]. Current mental health service users (N=40) where shown a series of recovery narratives and asked for qualitative and quantitative feedback on their impact. The short-term narrative impact model was developed through thematic analysis of the qualitative data. In the model, change is initiated through a recipient reflecting on their own experiences and then forming a connection through three mechanisms: comparing oneself with the narrative and/or narrator, learning about other people’s experiences, and experiencing empathy. The three mechanisms of connection lead to impact through the identification of change based on the narrative structure or the interpretation of change in the narrative content, both of which lead to the internalization of the interpretation by the individual. Factors moderating impact included clinical factors (eg, an inability to focus on the narrative due to symptomatology), personality (eg, long-term difficulty in connecting with others), and recipient preferences such as narrative modality.

The potential to create positive change means that using recorded recovery narratives as a mental health intervention is possible, but the possibility of negative impacts means that care is needed. The use of recovery narratives by health services has also been widely questioned. The critical theorist and activist collective Recovery in the Bin [39,40] and others [41] have argued that only very particular types of narratives that are perceived to be successful or acceptable are promoted by services. This can narrow the range of recovery templates available to recipients and potentially cause harm by limiting or negating recipients’ own ways of recovering [42]. Others have expressed concerns that recovery narratives might be co-opted, that is, used for purposes other than those intended by the narrator, and that this can sustain harmful structures such as poorly functioning health services [43] or deflect attention from systemic inequalities and social injustice [41]. These ethical issues must be carefully considered.

To inform the work of building an ethically defensible recovery narrative collection, a systematic review of decisions made in the curation of mental health recovery narrative collections was conducted [4]. The concept of curation draws on existing usage within the discipline of museum studies, where the work of curators has been extensively studied [44]. Curation is understood as both a purposeful and political act, with curators often engaging with artifacts or collections that are sensitive and challenging [45]. In total, 23 documents were identified, of which only one was a research publication. A significant knowledge gap was identified regarding curatorial decision making in relation to recorded mental health recovery narratives.

To address this gap, an interview study was conducted with 30 recovery narrative collection curators from 7 countries [24]. The qualitative analysis identified six categories of decisions made by curators comprising the VOICES (Values and motivations, Organization, Inclusion and exclusion, Control and collaboration, Ethics and legal, Safety and well-being) framework. It was concluded that collection curators have a great influence on how mental health and recovery issues are presented and understood and that recovery narrative collections can provide a mechanism for making collective rather than individual-level knowledge available.

#### NEON Intervention Development

This paper reports the process of developing the NEON Intervention, a new mental health intervention that provides access to recorded mental health recovery narratives. Digital health technologies [46] are increasingly being used in global mental health practice, motivated by challenges such as lengthy waiting lists for treatment [47], limited access to in-person mental health treatment in rural and remote communities [48-50], and the distress inherent in accessing in-person treatment for people experiencing social anxiety [51]. Digital health technologies are a crucial approach during the COVID-19 pandemic [52], when social connectedness is reduced [53].

The NEON Intervention provides access to the recorded mental health recovery narratives contained in the NEON Collection. The NEON Collection is a curated collection of recorded mental health recovery narratives. Narrators have given permission for their narratives to be used in the NEON Collection. Each narrative is characterized using a standardized inventory derived from the Recovery Narrative Conceptual Framework called the Inventory of Characteristics of Recovery Stories (INCRESE) [54].

Users of the NEON Intervention can access narratives in four ways:

1. Recommended: they can request the automated recommendation of recovery narratives in the NEON Collection. Requests are served by a recommender system [55], a term encompassing a family of algorithms designed to match digital media items to users. Recommender systems are frequently used in web-based digital media hosting services such as Spotify [56]. The design of the recommender system used in the NEON Intervention draws on the findings presented in this paper. Recommendations are informed by the narrative feedback provided after each narrative is received. For example, if accessing a recovery narrative makes a recipient feel more hopeful, they will receive more recommendations for recovery narratives with similar characteristics.
2. They can directly browse narratives in the NEON Collection by selecting tags of interest.
3. They can choose to be shown a randomly selected narrative.
4. They can rerequest a previously shown narrative.

The NEON Intervention is being developed and evaluated with three target groups: individuals who experienced psychosis, individuals with other mental health problems, and informal carers.

1. People who experience psychosis regularly use digital technologies such as social networks [57], and a systematic review of digital interventions for psychosis incorporating web-based, social media, and mobile technologies concluded that these approaches are acceptable, are feasible, and have the potential to improve outcomes [58]. Messages that promote hope are known to be recovery-promoting in psychosis [59], and hope is known to mediate potential psychosis recovery indicators such as increases in structured activity [60]. Accessing recovery narratives can reduce self-stigma, and self-stigma predicts low adherence to psychosocial treatments in patients with schizophrenia [61]. The effectiveness of the NEON Intervention for people who experienced psychosis will be evaluated in the definitive NEON Trial [27] (ISRCTN11152837).
2. The evidence reviewed earlier indicates that the benefits of accessing recorded recovery narratives are primarily transdiagnostic; therefore, the NEON Intervention may also be effective for people with nonpsychotic mental health problems. The effectiveness of the NEON Intervention for people with mental health problems other than psychosis will be evaluated in the definitive NEON-O Trial [27] (ISRCTN63197153).
3. With less strong evidence, recovery narratives may be helpful for informal carers, that is, family or friends of people with mental health problems, both for supporting their well-being and informing their understanding of the experiences of the person they care for. The feasibility of using the NEON Intervention with informal carers will be evaluated in the NEON-C Trial [27] (ISRCTN76355273).

### Aims and Objectives

The aim of this paper is to present selected items of knowledge developed during the intervention development process for the NEON Intervention and the study procedures for the 3 planned NEON trials. A detailed description of the final version of the NEON Intervention and of our chosen trial procedures is provided in our trial protocol [27]. In combination with our trial protocol, the knowledge presented in this paper will support the replication of the NEON Intervention, enable studies with other populations, and inform the development of new interventions using recorded recovery narratives. Some knowledge products (such as the curation principles described below) will have broader relevance to the design of interventions, integrating digital narratives about health and health problems.

The objectives of this study in the context of the development of the NEON Intervention by the NEON study are as follows:

1. Study objective 1: to develop the NEON Impact Model, a change model describing the impact of the NEON Intervention on recipients
2. Study objective 2: to identify appropriate curation principles for the NEON Collection of recorded recovery narratives used by the NEON Intervention
3. Study objective 3: to identify appropriate strategies for minimizing harm from the NEON Intervention
4. Study objective 4: to finalize narrative feedback questions for use by the recommender system
5. Study objective 5: to evaluate the acceptability and usability of an initial prototype of the NEON Intervention and associated trial procedures
6. Study objective 6: to identify features of intervention use relevant to trial planning

Study objectives 1-3 were addressed through three knowledge integration activities. These were methodical activities that drew on existing study expertise and knowledge to develop knowledge products underpinning the final version of the NEON Intervention evaluated in the NEON trials. Study objective 4 was initially addressed through research study 1, an experimental study in which quantitative feedback was provided by mental health service users who were shown items selected from the NEON Collection of recovery narratives. Study objectives 4-6 were addressed through research study 2, a feasibility evaluation of a prototype implementation of the NEON Intervention.

Knowledge produced through the three knowledge integration activities and 2 research studies is interlinked and is included in a single paper to provide a thorough account of the NEON Intervention development work. In selecting these five activities for inclusion, we focused on describing how human-computer interaction issues were successfully addressed in developing the NEON Intervention and trial procedures. This is in keeping with an accepted definition of human-computer interaction research as considering the broad personal and sociological context of technology usage [62]. Technical aspects of the development of the recommender system used in the NEON Intervention were also informed by the knowledge presented in this paper. These will be reported elsewhere.

The research reported here was conducted between September 2017 and October 2019. Ethical approval was received from the London - West London and Gene Therapy Advisory Committee Research Ethics Committee in advance (18/LO/0991). All participants provided informed consent, either in written or audio-recorded form. Appropriate consent was also collected for the inclusion of all narratives in the NEON Collection.

The methods and results for each of the five selected activities are described. As a result of research studies 1 and 2, refinements to the prototype implementation of the NEON Intervention used in research study 2 were made. These refinements are described in the Results section. We reflect on the broader implications of our findings in the Discussion section.

## Methods

### Knowledge Integration Activity 1: Development of the NEON Impact Model

This knowledge integration activity developed the NEON Impact Model, describing how recorded recovery narratives improve outcomes. The modeling of change is recommended in the UK Medical Research Council guidance on the development and evaluation of complex evaluations [63].

The 3 completed NEON studies on impact [1,36,38] were synthesized by the NEON research team to produce a change model comprehensively describing how health-related outcomes might be produced by receiving recorded recovery narratives. The short-term narrative impact model was selected as the theoretical foundation of the integrated change model because it was produced by an experimental study considering only recorded recovery narratives. To incorporate longer-term impacts, relevant components from the Narrative Impact Conceptual Framework and the long-term narrative impact model were integrated if they described health-related change. Informed by the biomedical principle of nonmaleficence [64], negative outcomes identified in other eHealth studies [65] were added if they might be produced through use of the NEON Intervention, to inform the design of harm minimization strategies or trial procedures. *Moderators* and *forms of learning* were present across all underpinning studies; hence, comprehensive lists were synthesized by merging similar items and grouping them into superordinate categories.

### Knowledge Integration Activity 2: Identification of Curation Principles

This knowledge integration activity identified appropriate curation principles for the NEON Collection of recovery narratives. Implementing these principles ensured that the NEON Collection addresses all safeguarding, ethical, legal, clinical, and technological challenges associated with storing and using recovery narratives.

#### Measures

The INCRESE is a 77-item, researcher-rated standardized tool to identify manifest and latent characteristics of recorded mental health recovery narratives [54]. Categories in INCRESE comprise narrative eligibility, narrative mode, narrator characteristics, narrative characteristics, and narrative content. In total, 71 items characterize manifest content (eg, narrator gender, narrator diagnosis, content requiring content warnings, types of turning point, specific topics such as family, education, or work), and 6 characterize latent content (stage of recovery, genre, positioning, tone, relationship with recovery, and trajectory). Specific attention was paid to the issue described earlier, relating to the importance of diverse recovery templates, so items and rating categories included *uses a nondiagnostic framework*, *recovery despite services*, *recovery outside services*, *narrator rejects the concept of recovery as used in mental health services*, and *circular trajectory*. Different rating scales are used for different sections, for example, present or mainly positive or mainly negative for specific narrative content and escape or endurance or endeavor or enlightenment for genre. No summary scores were produced.

#### Procedures

All elements of the design of the NEON Collection were explored using the VOICES typology of curatorial decisions as an organizing framework [24]. The research team sought advice on specialist issues from relevant experts, for example, legal advice was obtained in relation to intellectual property and references to third parties in recorded recovery narratives. To ensure that the curation principles for the NEON Collection were informed by a lived experience perspective, rather than reflecting solely research and clinical priorities, three consultation workshops with the NEON Lived Experience Advisory Panel (LEAP) were held between October 2017 and June 2018. The NEON LEAP comprises an independent chair and 10 members with an interest in recovery narratives and personal experience of mental health problems and services either directly or as family members. Workshop agendas were co-produced between a subgroup of the NEON LEAP and the research team, who met at least two weeks before each workshop to finalize the agenda and precirculate preparatory materials in line with best practices [66]. Workshops addressed issues presenting complex ethical challenges, such as inclusion and exclusion criteria, appropriate approaches to anonymization, withdrawal of narratives, and procedures for processing third-party requests for narrative withdrawal. Each workshop was attended by approximately 15 participants, comprising all NEON LEAP members and several research team members. Each workshop involved facilitated whole-group and subgroup discussions to systematically explore curatorial issues from a lived experience perspective. The meetings were minuted in detail. LEAP members were paid GB £150 (US $210) plus expenses per workshop. This work informed the ethics application enabling intervention development work. This was approved by the UK Health Research Authority to govern the long-term management of the NEON Collection.

A key challenge identified through work with LEAP was the selection of appropriate inclusion and exclusion criteria for narratives. To refine these criteria and to ground them in practical challenges, a preliminary collection of 100 recovery narratives was assembled from publicly accessible resources and with the permission of the collection curator or narrator as required. The NEON Collection Steering Group (CSG) comprised 4 NEON LEAP members and 2 NEON researchers. Each narrative was coded by at least one researcher against the proposed inclusion and exclusion criteria and classified as either clearly included, clearly excluded, or not clear. Narratives coded as not clear were referred to the NEON CSG for a final decision. The CSG decided on inclusion and identified any necessary clarifications regarding the wording and interpretation of the inclusion or exclusion criteria. All decisions were minuted, and the current inclusion and exclusion criteria were updated on the NEON Collection web page [67].

Curation involves a careful review of narratives, which can create a significant emotional burden [36]. Furthermore, narratives that met the inclusion criteria were coded using INCRESE before inclusion in the NEON Collection, which involved a detailed assessment of a large number of narratives. In an unplanned add-on study, a feedback questionnaire was completed by INCRESE coders, covering the emotional impact of characterizing narratives and personal strategies for well-being [54]. These questionnaires were analyzed to identify general principles to support curator well-being. A facilitated discussion was then held involving all CSG members or NEON researchers who had coded at least 30 narratives. Participants reflected on personal well-being strategies, considered the questionnaire analysis, and identified the principles of well-being. The consensus recommendations were minuted.

#### Analysis

To synthesize knowledge on the most critical decisions taken during this process, a document analysis [68] was conducted on the minutes of NEON LEAP consultation meetings (n=3), the approved research protocol, minuted recommendations produced by CSG, the NEON Collection page on the NEON website, and minuted recommendations from the workshop on coder well-being. The documents were imported into NVivo (QSR International; version 11). The text describing a decision or the rationale for the decision was identified and summarized. Decisions were then organized into six predefined categories of the VOICES framework of narrative curation.

### Knowledge Integration Activity 3: Identification of Harm Minimization Strategies

This knowledge integration activity identified appropriate strategies for minimizing harm from the NEON Intervention.

#### Participants

Strategies were developed through consultation between the NEON research team, the NEON LEAP as described in knowledge integration activity 2, and the NEON International Advisory Board, which comprises 7 experts in research and intervention development around recovery narratives, eHealth interventions, peer research, and mental health recovery.

#### Procedures

An iterative approach was used to identify, implement, and refine candidate harm minimization strategies. The NEON Impact Model was used as a foundation for shaping harm minimization strategies, and an evolving design rationale [69] explaining essential decisions and why they were made was created. This was expressed in the form of a draft protocol for the 3 NEON trials. An early draft of the trial protocol was discussed with NEON International Advisory Board members, and the prototype developed for research study 2 was then discussed at two meetings between a subgroup of NEON LEAP and NEON researchers. These consultations led to protocol and prototype enhancements. The prototype was considered at a NEON LEAP meeting, and further enhancements were made.

One area in which efforts were made to be guided by research was in relation to content warnings, also known as trigger warnings, defined in an education context as “offering prior notification of an educational topic so that students may prepare for or avoid distress that is automatically evoked by that topic due to clinical mental health problems” [70]. The research team conducted a nonsystematic narrative review of research on content warnings [27]. The review found limited and conflicting evidence, primarily drawn from educational or trauma treatment settings [70-75]. No directly relevant evidence concerning the impact of content warning in relation to mental health recovery narratives was identified. NEON is a health service–funded study, so it was decided to follow the standard clinical practice of giving content warnings on the basis of the ethical principle of nonmaleficence.

### Research Study 1: Narrative Feedback

The aim of research study 1 was to evaluate an initial set of narrative feedback questions designed for use in the recommender system. This contributed to the selection of a final set of narrative feedback questions, in keeping with study objective 4. The specific objectives of research study 1 were to identify floor and ceiling effects in narrative feedback, to describe variability among participants, and to examine whether the order of presentation affected response. The latter was included because it might indicate fatigue in the repeated provision of feedback. The analysis used quantitative narrative feedback data collected in the short-term narrative impact model study [38] but only qualitative data from this study have previously been reported.

#### Participants

Eligible participants were people with current mental health concerns, using statutory mental health services, aged above 18 years, able to provide informed consent, and fluent in English. Individuals who were experiencing a crisis or who were otherwise unable to participate in the research were excluded.

#### Setting

Participants were recruited from statutory mental health services within a health care trust in the East Midlands of England.

#### Measures

The Herth Hope Index (HHI) is a 12-item measure of hope adapted from the Herth Hope Scale with adequate psychometric properties [76]. The HHI score ranged from 12 (low hope) to 48 (high hope).

#### Procedures

A subset of 30 narratives were assembled from the NEON Collection by 2 researchers. Narratives were purposively selected to maximize the variation in modality, narrator diversity, and length. Modality was chosen because multimedia use in educational settings has been shown to increase the depth of learning in students [77], suggesting that this may promote engagement and cater to different learning styles within individuals. Narratives with a substantial range of modalities are available in the public domain, and the use of multimedia may also promote the inclusiveness of individuals who experience disabilities or who may have difficulty comprehending a specific mode of media, for example, due to dyslexia. The selected narratives were diverse in narrator age, gender, and ethnicity, given the evidence from the NEON Impact Model that sociodemographic characteristics can influence connection. Finally, to vary the cognitive demands of participants, the chosen narratives were different in length. Text narratives ranged from half a page to 3 pages, video narratives ranged from 1 to 5 minutes, and audio narratives ranged from 2 to 3 minutes. On the basis of a pilot study protocol, it was estimated that, on average, participants would take no longer than 10 minutes to read, watch, or listen to a narrative. A total of 30 narratives were selected, comprising 15 texts (poems and prose text), 10 videos, and 5 audio-based narratives.

The study was promoted as an investigation of narrative impact through social media, advertisements within services (eg, posters and newsletters), and by clinicians and managers from Improving Access to Psychological Therapy Services, community forensic services, locality mental health teams, and recovery colleges. Both clinician referrals and self-referrals to the study were accepted. Potential participants were given a participant information sheet by their clinician or researchers. Interested participants then contacted the researchers or gave their clinician permission to pass on their contact details. The researchers assessed eligibility, and informed consent was obtained before the interview. Interviews took place in a university or clinical setting.

Each participant took part in a two-hour research session, for which they were offered GB £20 (US $28) plus expenses. After providing informed consent, the researcher completed a participant characterization form from the verbal responses provided by the participant. The characteristics recorded included age, gender, preference for narrative modality, current diagnosis, and hope (HHI). Each participant then sequentially received randomly selected narratives, discontinuing when the participant indicated that they wished to stop. If individuals expressed a preference for narrative modality, then only narratives consistent with their preferences were considered. After accessing each narrative, the participants provided narrative feedback by rating three questions: How connected to the story did you feel? How connected to the narrator of the story did you feel? How hopeful did the story make you feel? Each response was rated on an 11-point scale (rating: 0-10). The labels for the two questions on connection were as follows: 0, extremely disconnected; 2, somewhat disconnected; 5, neither connected nor disconnected; 7, somewhat connected; and 10, extremely connected. The labels for the question on hopefulness were as follows: 0, extremely pessimistic; 3, somewhat pessimistic; 5, neither hopeful nor pessimistic; 7, somewhat hopeful; 10, extremely hopeful.

#### Analysis

Quantitative analysis was conducted using SPSS statistics (IBM; version 25) and STATA SE (version 16). The significance level was set at *P*=.05. Narrative feedback responses were grouped by question and then tested for nonnormality using the Kolmogorov-Smirnov test. No evidence of nonnormality was found; therefore, parametric tests were used.

Floor and ceiling effects and variability across participants were inspected by creating box-and-whisker plots. Outliers were defined as cases that fell 1.5 times above or below the IQR, and extreme outliers were defined as cases falling 3 times above or below the IQR. The impact of order of presentation was evaluated by creating a derived variable called presentation order (first, second, third, fourth, fifth, etc) and conducting a linear multilevel regression with each narrative feedback question as a dependent variable. This was because all participants accessed at least four narratives but not all accessed five or more.

### Research Study 2: Feasibility Evaluation

The aim of research study 2 was to evaluate the feasibility of using the NEON Intervention and associated trial procedures with people with experience of mental health problems. The specific objectives were to finalize narrative feedback questions (study objective 4), to evaluate the acceptability and usability of an initial prototype of the NEON Intervention and associated trial procedures (study objective 5), and to identify features of intervention usage relevant to trial planning (study objective 6).

#### Participants

Eligible participants were people with current mental health concerns; using statutory mental health services; aged ≥18 years; having the capability, with support if needed, to interact with a web-based intervention; having access to a computer or smartphone with an internet connection at home, in a community venue, or through a health service venue; who are able to provide informed consent; and who are fluent in English. Individuals who were experiencing a crisis or who were otherwise unable to participate in the research were excluded.

#### Setting

Participants were recruited from statutory mental health services within a health care trust in the East Midlands of England.

#### Procedures

Research participants were recruited through a single health care trust in England, using three strategies. First, posters and leaflets were placed in health services and community venues. Second, participants were recruited through a direct approach by clinical support officers who attended health care clinics. Third, communication was sent by the research team to prior NEON research participants who provided ongoing consent to contact.

A prototype web-based NEON Intervention was developed as an interactive platform for implementing strategies and allowing the features to be used by others to inform refinements. The platform integrated the NEON Intervention with selected web-based trial procedures, such as eligibility testing and web-based consent processes. All components of the NEON Intervention were implemented apart from the recommender system, and the baseline measures component was implemented in reduced form, that is, with fewer measures. Data usage was automatically logged, including the device type used to access the intervention, time between key trial processes (informed consent, baseline measure completion, randomization, first narrative presentation, etc), frequency, and context of usage of specific features (eg, “I’m upset” or “Get me out of here”).

Participants attended a 2-hour baseline interview at a research site, for which they were offered GB £20 (US $28) payment plus expenses. They worked through a series of tasks using a prototype. The tasks were to read the web-based participant information sheet; provide web-based consent through an informed consent form; register a NEON Intervention account using an email address; complete a web-based participant demographics form; complete the 12-item Manchester Short Assessment of Quality of Life [33], which is the primary outcome; complete the 10-item CORE-10 (Clinical Outcomes in Routine Evaluation) measure of problems, functioning, and risk [78], which is a secondary outcome; access an initial random narrative and provide narrative feedback; use the NEON Intervention to select an additional narrative and provide narrative feedback using the same three questions and rating scale as in study 3; and if time allowed, to explore the interface, including features such as the “About me,” “I’m upset,” and “Get me out of here” buttons.

During these tasks, participants were asked to verbalize their thoughts using a think-aloud protocol [79]. The researcher did not guide the participant, although occasionally the researcher assisted if the participant was stuck and could not progress despite substantial effort. The planned exception was that the researcher would proactively assist a participant who indicated that they normally needed support in using a computer system, but this did not occur in practice. After the tasks were completed, the participants were asked two trial procedure questions: Would you be happy to receive payment for trial participation through online vouchers? What might prompt you to use the intervention after your first use of it? The interview was recorded, and the researcher made field notes on the observations.

Following the baseline interview, participants were given unconstrained access to the web-based prototype for 28 days, during which time their usage was automatically logged. A follow-up interview was then conducted at a research site, and participants were paid GB £20 (US $28) plus expenses. A summary of their recorded usage of the intervention was prepared, showing the number of narratives accessed and rated, number and date of log-ins, and proportion of log-ins on mobile devices (smartphones or tablets) or computers (laptops or desktops). At the follow-up interview, participants were shown their usage and asked to discuss notable features, such as periods of high or low usage. Data usage summary sheets as a form of data visualization are a standard approach to support reflection on computer system usage [80]. If a system or system feature is underused, the discussion of usage data can differentiate whether this was due to (1) the system creating immediate positive change requiring no further engagement, (2) periods of planned technological disconnection such as holidays, or (3) malfunction, dislike, or poor usability of the system or feature [81].

The prototype was also discussed at a NEON LEAP meeting and minutes were taken. NEON LEAP members were subsequently given access to the prototype and provided written feedback.

#### Analysis

To meet study objective 5, a list of prototype features was created. For each feature, feedback from baseline and follow-up interviews and NEON LEAP feedback were synthesized. Short narrative summaries of responses to the two trial procedure questions were produced, and the baseline field notes were analyzed thematically. Each element of feedback was categorized as acceptable, unacceptable, usable, less usable, and other. Categories of unacceptable and less usable identified features that most needed improvements. Categories of acceptable and usable were included to indicate variations in response across the cohort, for example, to identify whether there were features that were acceptable to some but unacceptable to others. Feedback in the other category was reviewed individually.

To meet study objectives 4 and 6, descriptive analyses were conducted to describe participant demographics, frequency and route of access, frequency of narrative feedback, and frequency and length of use of the NEON Intervention.

## Results

### Knowledge Integration Activity 1: NEON Impact Model

**Figure 1:**
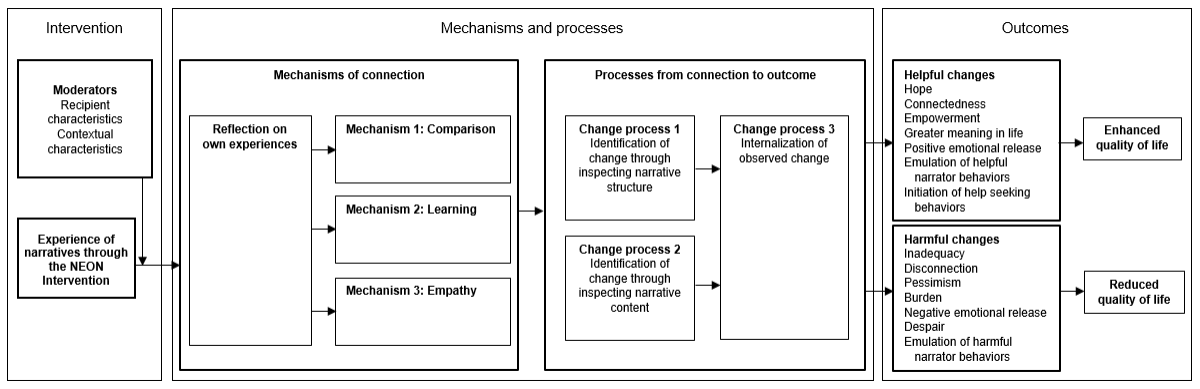
Narrative Experiences Online Impact Model linking experience of narratives to outcomes. NEON: Narrative Experiences Online.

The NEON Impact Model, describing the processes by which engaging with a recorded recovery narrative can create change, is shown in Figure 1.

In the NEON Impact Model, the impact of a recovery narrative is moderated by the characteristics of the recipient and the context in which they receive it. A complete list of recipient and contextual moderators synthesized from the source studies is shown in Table 1.

In the NEON Impact Model, one way for a narrative to make an impact is through the recipient learning something from the narrative (mechanism 2 in Figure 1). The types of learning synthesized from the source studies are shown in Textbox 1.

The NEON Impact Model informed (1) the design of the NEON Intervention, including the narrative feedback questions and guidance about using the intervention, and (2) the trial process evaluation.

**Table 1:** Moderators of the impact of recovery narratives.

Moderator		Direction of influence
**Recipient characteristics**		
	Recipient reports a long-term inability to connect with others.	Reduced impact
	Recipient has experienced a recent event perceived as distressing.	Reduced impact
	Recipient is experiencing a mental health crisis.	Reduced impact
	Recipient has beliefs, values, or attitudes contradicting those of the narrator.	Reduced impact
	Recipient is experiencing mental health problems that disrupts information processing (such as hearing voices).	Reduced impact
	Recipient perceives the content of the narrative to be emotionally challenging.	Reduced impact
	Recipient experiences difficulties in comprehending the form of the narrative (eg, if the narrative is presented as a poem).	Reduced impact
	Recipient perceives the narrative or narrator to be inauthentic.	Reduced impact
	Recipient perceives the narrative or narrator to be authentic.	Increased impact
**Contextual characteristics**		
	Recipient has access to a private space to access challenging narratives.	Increased impact
	Recipient has access to a mental health worker who supports processing.	Increased impact

**Types of learning from accessing recovery narratives.**

**Learning About Mental Health**

- How others experience a mental health condition
- Alternative conceptualizations of mental health problems
- The impact of mental health problems on others (eg, carers)
- New coping strategies to enhance daily living

**Learning About Recovery**

- Recovery is possible
- Specific recovery strategies that have helped others
- Barriers to recovery that others have experienced
- Differing beliefs and values that have supported recovery
- How to manage treatment and make best use of services

### Knowledge Integration Activity 2: Curation Principles

The NEON Collection curation principles are listed in Textbox 2. These have been implemented in full to create, manage, and use the NEON Collection.

**Curation principles for the Narrative Experiences Online Collection.**

**Curation Principles**

VOICES (Values and motivations, Organization, Inclusion and exclusion, Control and collaboration, Ethics and legal, Safety and well-being) domain 1: values and motivations of the Narrative Experiences Online (NEON) Collection

- Purpose: the primary purpose of the NEON Collection is to provide benefits to recipients.
- Mission: the NEON Collection will seek for heterogeneity of narrative content, form, and narrator demographics and to be as large as possible. Greater heterogeneity and size increases the chance of a recipient finding someone like them or a story like theirs and hence experiencing the helpful outcomes outlined in Figure 1. Insufficient heterogeneity and size risks a recipient failing to find someone like them and hence feeling more disconnected from others.
- Less hopeful narratives: the NEON Collection will include some narratives where adversity and struggle are the dominant themes. Although these narratives are less regularly used by health services, recipients experiencing profound distress may find it easier to connect with such narratives.

VOICES domain 2: organization of the NEON Collection

- Donation from existing collections: narratives can be donated to the NEON Collection by organizers of existing collections but only if the collection organizer confirms that appropriate consent has been obtained and only if the narrative is already public. Appropriate consent means either the collection organizer has previously collected consent to enable reuse or has obtained consent from individual narrators to donate their narrative to the NEON Collection. Details of the source collection will be retained and may be displayed to recipients to help them understand the context of the narrative.
- Donations from individuals: narratives can also be offered to the NEON Collection by individual narrators, even if they have not been published.
- Role of the curator: NEON researchers will assess inclusion of narratives, with involvement of the Collection Steering Group (CSG), the NEON Chief Investigator, or a legal expert where uncertainty exists around specific exclusion criteria. Diversity in the NEON Collection will be monitored to identify underrepresented groups to be targeted for narrative donation. Curators of the NEON Collection will not edit narratives, which will be displayed as close as possible to their original form.

VOICES domain 3: inclusion and exclusion of narratives in the NEON Collection

- Decision-making process: decisions on inclusion will be made with reference to formal current inclusion and exclusion criteria. These criteria will be publicly available to ensure transparency. Numbers of narratives considered, included, and excluded will be published for transparency. If stories link to external material, then the contents of this material should not be considered when deciding inclusion, as it may change. Donors (either individual narrators or collection organizers) will be informed if their narrative is not included, and there will be an appeal process.
- Inclusion criteria: a narrative is includable in the NEON Collection if all of the following criteria are met—(1) it includes elements of adversity or struggle that relate to mental health problems, broadly defined; (2) it includes descriptions of strength, success, or survival, as defined by the narrator or identifiable by a third party; (3) it refers to events or actions over a period (including either external events or internal mental events); (4) it is told by an individual with experience of mental health problems and recovery; (5) where language is used, the narrative is mainly in English or, if translated, the translation needs to be provided or approved by the narrator; (6) the story is provided in a digital file, the story is provided in a format that can easily be converted into a digital file, or the story is hosted on an existing webpage, the URL to the webpage is permanent, and the page does not contain links that would enable navigation to another page; or (7) consent to use the narrative in perpetuity (other than if the narrative is withdrawn) has been obtained from the narrator, from the owners of an existing collection who have previously collected consent from their donors that is broad enough to allow for reuse, or from the owners of an existing collection who have collected individual consent from their donors for usage in the NEON Collection.
- Exclusion criteria: a narrative is excluded from the NEON Collection if any of the following criteria are met—(1) it is presented as fictional; (2) it is told by anyone other than the individual experiencing mental health problems and recovery (such as a carer or journalist); (3) (for video and audio stories) the quality of recording is so low that the story is very difficult or not possible to understand; (4) it is split across multiple files or modalities or uses a multimedia approach that cannot easily be integrated into a single file; (5) it contains descriptions of potentially harmful behaviors in sufficient detail as to be likely to encourage imitation; (6) it indicates that the narrator has engaged in an undisclosed, serious criminal activity; (7) the narrator is a child or appears to be a child, unless it has been confirmed that the narrator is now an adult and has provided consent for a childhood story to be shared; (8) it contains hate speech; (9) it provides information about a third party that might reasonably lead to harm being caused to the third party such as providing directly identifying information about someone accused of abuse; (10) it includes sensitive personal information about individual third parties, unless the third party has already made this information public, for example, by publishing their own recovery story, or unless the third party is no longer alive. A story includes sensitive information about a third party if it clearly reveals their political or religious beliefs, mental or physical health conditions, sexual orientation or behaviors, or any offences committed or alleged to have been committed by them; (11) it reveals the adoption status of a third party, unless the third party has already made this information public; or (12) it raises any other unforeseen concerns, in which case this list of reasons for exclusion may be updated. Exclusion criterion 9 is included for predominantly legal reasons: the NEON Collection is hosted in the European Union (EU) and hence subject to the EU General Data Protection Regulations on personal information.
- Resolution of uncertainty: when making an assessment, there will be a bias toward the inclusion of a narrative. For example, inclusion criteria 2 is met if any rater can see strengths, successes, or survival in a narrative. If NEON researchers are uncertain whether a narrative meets all the inclusion criteria or exclusion criterion 1, 2, or 3, a final decision will be made by CSG. If the uncertainty is about exclusion criterion 4, 5, 7, 11, or 12, a final decision will be made by the NEON Chief Investigator. If the uncertainty is about exclusion criterion 6, 8, 9, or 10, an opinion will be sought from a legal representative approved by the study sponsor. Some forms of uncertainty can be resolved by asking the narrator for a short addendum to contextualize the narrative, but narrators are not required to submit this or may not be contactable, and hence final decisions may need to be made without it.

VOICES domain 4: control and collaboration around the NEON Collection

- Oversight: for the duration of the NEON program, the study sponsor will act as an auditor and may examine records relating to narrative consent. If use of the NEON Collection continues beyond the end of the study, an equivalent authority needs to be in place and approved by the study sponsor.
- Archiving and reinstatement: the NEON Collection can be temporarily archived (eg, at the end of the NEON program) and withdrawal requests cannot be met while it is archived. It can only be reinstated from the archive if a body with an equivalent status to a study sponsor is identified.
- Information about approvals: details of legal and ethical approvals for the NEON program and the NEON Collection will be displayed whenever narratives from the NEON Collection are used.

VOICES domain 5: ethical and legal considerations for the NEON Collection

- Documentation of consent: if a collection organizer wishes to offer narratives to the NEON Collection, they must confirm in writing that consent has been provided. This confirmation will be stored for audit purposes.
- Rights of collections: collection organizers have the right to withdraw any narratives that they have donated.
- Rights of the narrator: accepted narrators have a right to inclusion and publication of a short addendum. They might use this to illustrate how their life has changed since they created their narrative or to contextualize what was happening in their life at the time they wrote their narrative. All narrators have a right to withdraw a narrative. They can request withdrawal through a collection organizer if the narrative was donated from an existing collection or directly through the NEON Collection in all cases.
- Rights of third parties: third parties can request withdrawal, for example, if they assert that a narrator did not have capacity when they submitted a narrative, and each request will be individually assessed by the NEON CSG. To protect the right of narrators to have their story told, third parties do not have an automatic right to withdrawal.
- Processing of withdrawal requests: all narratives will be given a unique ID to aid withdrawal requests. Since some withdrawal requests may be malicious, such as an attempt by someone who is not the narrator to withdraw the narrative without due cause, in order to protect the rights of narrators and the existence of the NEON Collection, proof of identity may be required. Low-burden mechanisms will be provided to establish identity.
- Assertion of copyright breach: individuals can assert that a narrative has breached their copyright, and assertions of copyright theft will be processed in accordance with the European e-Commerce Directive 2000/31/EC [82].
- Expectations on recipients: to access narratives in the NEON Collection, a user must register an account and commit to not copying any material. This is because some individuals have donated narratives that are not published elsewhere.

VOICES domain 6: safety and well-being

- Safety of narrators: the NEON Collection will not edit or anonymize narratives that have been submitted because stories can be an economic and social resource for some narrators and because this may have intellectual property implications for the NEON program. If an individual donates their narrative to NEON, they will be provided with information about how the narrative will be used and encouraged to think about consequences of revealing their identity in a narrative, allowing them to make an informed choice about whether to be identifiable. They can submit identifying metadata (such as a story title that includes their name) if they wish.
- Safety of curators: when assessing narratives for inclusion, curators have the right to disengage from a narrative that distresses them, either temporarily or permanently, without providing a reason.

### Knowledge Integration Activity 3: Harm Minimization Strategies

Several harm minimization strategies were identified and implemented in the NEON Intervention.

#### Strategy 1: Informed Consent

Potential participants were informed through a web-based participant information sheet about the potential harmful impacts listed in the NEON Impact Model (Figure 1). This allowed participants to make an informed choice about whether to participate in the study.

#### Strategy 2: Reflecting on Self-management

Before receiving the first narrative, participants received brief advice on how to handle difficult emotional responses to narratives. They were then asked to record self-management strategies that they find helpful to use when upset. This encouraged participants to apply their self-management strategies if needed when using the NEON Intervention. They can change the recorded strategies in the “About me” section of the intervention.

#### Strategy 3: Dealing With Emotional Distress

Participants were encouraged to use the “I’m upset” button if they become upset while using the NEON Intervention. This button is prominently available on all intervention pages and opens a webpage, providing access to four resources:

- A reminder of any self-management strategies they previously recorded.
- Information about national helplines in England: Samaritans, Mind, The Mix, Elefriends, Big White Wall, Saneline, and Rethink Mental Illness.
- Evidence-based self-management resources organized into categories such as express yourself creatively, labeling your feelings, mindfulness, self-soothing, meditation, breaking up triggers, and distraction.
- Information about contacting a general practitioner, local mental health services, and the National Health Service (NHS) urgent and emergency care hotline.

#### Strategy 4: Content Warnings

INCRESE rates five sensitive issues: abuse or sexual violence, loss of life or endangerment to life, self-harm including eating disorders, violence or aggression and injustice, and prejudice and discrimination. As interpretation is needed in relation to some of these, compromising interrater reliability, INCRESE items relating to content warnings only will be independently rated a second time by a different rater. If either rater identifies a content warning as relevant, it will be included in the final INCRESE rating. If the INCRESE rating of a narrative indicates it deals with any of the five content warning issues, then all relevant content warnings are displayed before the presentation of the narrative. The participant must actively select to proceed to be presented with the narrative.

#### Strategy 5: Proactively Blocking Categories of Narratives

Participants have the ability to block categories of narratives. They can be blocked based on the modality or content. For modality, they have the option to block up to three of the four categories: text, audio, moving images, and static images. For content, they have the option to block any of the five INCRESE content warning categories: abuse or sexual violence, loss of life or endangerment to life, self-harm including eating disorders, violence or aggression and injustice, and prejudice and discrimination. Narratives in each blocked category are not considered for future presentation. The “About me” section of the website contains the option to unblock previously blocked categories of narratives.

#### Strategy 6: Reactively Blocking Individual Narratives

If a participant finds a narrative distressing, they can block it during or after its presentation. The narrative will be immediately hidden from them and will not be considered for future presentation. The “About me” section contains the option to unblock previously blocked individual narratives.

#### Strategy 7: Easy Exit

A button labeled “Get me out of here” is prominently provided throughout the intervention, which when pressed goes to a neutral webpage. This can be used if a participant feels overwhelmingly distressed and wants to quickly leave the intervention or if the interface is being accessed in a public setting and a participant does not want others to know about their usage.

### Research Study 1: Narrative Feedback

The clinical and sociodemographic characteristics of the 40 participants included in this study are shown in Table 2.

**Table 2:** Characteristics of participants of study 3 (N=40).

Characteristics			Participants
Gender (female participant), n (%)			24 (60)
Age (years), mean (SD)			44.4 (16.7)
Modality preference indicated, n (%)			28 (70)
Herth Hope Index, mean (SD)			31.1 (5.3)
**Diagnosis, n (%)**			
	Mood disorder	15 (38)	
	Schizophrenia or other psychosis	9 (23)	
	Bipolar disorder	7 (18)	
	Personality disorder	8 (20)	
	Other	1 (3)	
**Self-rated recovery trajectory, n (%)**			
	I am recovered	1 (3)	
	I am living well	4 (10)	
	I am making progress	18 (45)	
	I am surviving day to day	17 (43)	

Participants provided feedback on a total of 281 narratives, with a median of 7 randomly selected narratives (range 4-14) accessed per participant from a pool of 30 narratives.

**Figure 2:**
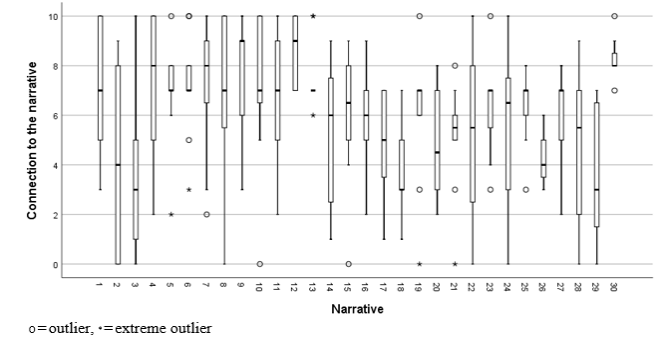
Ratings by participants (N=40) of connection to the narrative.

For the 281 ratings of narratives, the mean ratings for connection to the narrative (mean 6.03, SD 2.77), connection to the narrator (mean 5.76, SD 2.80), and hope (mean 5.31, SD 2.63) indicated that the narratives had, on average, a neutral to small positive impact on participants in terms of connection and hope, as the chosen rating scales used a value of 5 to indicate a neutral impact. The distribution of narrative connection ratings for each of the 30 narratives is shown in Figure 2.

**Figure 3:**
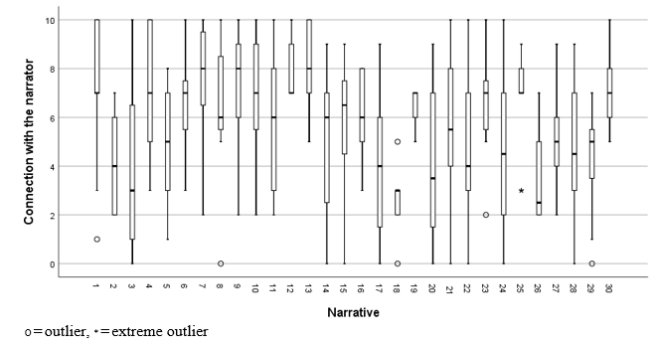
Ratings by participants (N=40) of connection with the narrator.

The distribution of narrator connection ratings for each narrative is shown in Figure 3.

**Figure 4:**
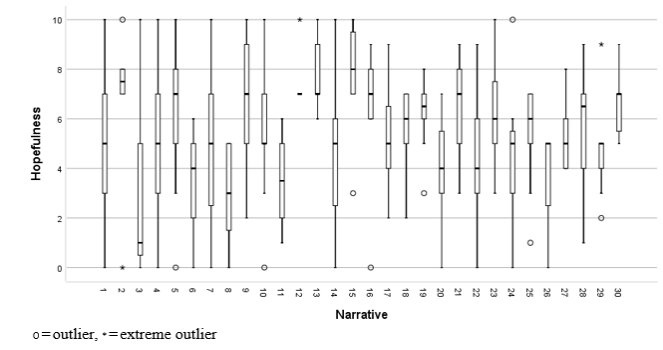
Ratings by participants (N=40) of hopefulness for each narrative.

The distribution of hopefulness ratings for each narrative is shown in Figure 4.

There were no apparent floor or ceiling effects in narrative feedback. Narratives 12 (range 7-10) and 13 (range 6-10) were hope-promoting for all participants in the sample, and narrative 15 had the highest median hope rating of 8.0 (IQR 7-9.8). However, hope ratings were more widely distributed for the other 28 narratives, which were hope-promoting for some participants but hope-reducing for others. The order of presentation did not predict any of the three narrative feedback ratings, and hence, there was no evidence of fatigue effects.

Collectively, these results provide no rationale for discarding the candidate narrative feedback questions evaluated in this study, but they indicate that narrative feedback might be used to tailor recommendations of narratives to individuals, given substantial variability among participants on feedback provided.

### Research Study 2: Feasibility Testing

The clinical and sociodemographic characteristics of the 25 participants included in this study are shown in Table 3.

**Table 3:** Characteristics of participants of study 4 (n=25).

Characteristics		Participants
Age (years), mean (SD)		39.3 (11.6)
**Ethnicity, n (%)**		
	Asian	4 (16)
	White	21 (84)
**Occupation, n (%)**		
	Sheltered employment	1 (4)
	Employed	9 (36)
	Unemployed	8 (32)
	Retired	3 (12)
	Training or education	4 (16)
**Living situation, n (%)**		
	Alone	8 (32)
	With others	17 (68)
**Education, n (%)**		
	No qualification	2 (8)
	O-levels, General Certificate of Secondary Education, or equivalent	3 (12)
	A-levels, National Vocational Qualification, or equivalent	5 (20)
	Degree-level qualification	11 (44)
	Higher degree–level qualification	4 (16)
**Service contact, n (%)**		
	No contact with any National Health Service	0 (0)
	Contact with my general practitioner only (self-reported)	1 (4)
	Contact with my general practitioner and with IAPT^a^	1 (4)
	Contact with my general practitioner and a specialist mental health team	23 (92)
**Duration of service support in years**		
	Mean (SD)	13.88 (9.45)
	Median (IQR)	10 (7-20)
Has ever been an inpatient, n (%)		10 (40)
**Current diagnosis, n (%)**		
	Schizophrenia or other psychosis	5 (19)
	Bipolar disorder	9 (33)
	Mood disorder	4 (15)
	Other (included ADHD^b^, posttraumatic stress disorder, personality disorder, and autism)	9 (33)
**MANSA^c^ score**		
	Mean (SD)	4.13 (1.01)
	Median (IQR)	4.5 (3.58-4.67)
**CORE^d^-10 score**		
	Mean (SD)	18.84 (9.11)
	Median (IQR)	20 (13-23)

^a^IAPT: Improving Access to Psychological Therapies, a mental health treatment program provided by the National Health Service in England

^b^ADHD: attention-deficit/hyperactivity disorder.

^c^MANSA: Manchester Short Assessment of Quality of Life.

^d^CORE: Clinical Outcomes in Routine Evaluation.

Follow-up interviews were conducted with 22 participants. One nonattending participant experienced a serious adverse event confirmed as unrelated to the study, and two were unavailable in the follow-up interview period due to significant life events.

#### Study Objective 5 (Acceptability and Usability)

Feedback during the baseline interview was broadly positive, and no participant indicated that trial procedures or the NEON Intervention as a whole was unacceptable. Some participants commented that the design of the site used a large amount of blue and white, giving it the feel of a UK-based NHS website, which had negative associations for some.

A summary of the feedback specifically relating to trial procedures, which was rated as unacceptable or less usable, is shown in Textbox 3.

**Identified issues relating to Narrative Experiences Online trial procedures.**

**Identified Issues**

- Participant Information Sheet (PIS): the PIS was too long. The focus on information about possible harms felt excessively negative and off-putting. A plain English summary would be helpful.
- Informed consent form (ICF): recording initials in the ICF created anxiety about confidentiality for one participant.
- Demographics form: the small number of categories for ethnicity was perceived as discriminatory. Some participants wanted to know whether the demographic information would be used in the recommender system.
- Manchester Short Assessment of Quality of Life (MANSA): some items (eg, “How satisfied are you with your sex life?”) were personal, embarrassing, or uncomfortable to fill in, especially with a researcher present. Submitting the form and then pressing the back button displayed a blank MANSA form, so it was not clear whether the data were successfully submitted.
- CORE-10: several participants felt distressed by a question on suicidality, and one suggested the “I’m upset” button should be available from this form onward. A suggestion was made that items be rated 1-10 instead of 1-5 and not to reverse-score some items.

A summary of the feedback relating to the NEON Intervention is shown in Textbox 4.

**Identified issues relating to the Narrative Experiences Online Intervention.**

**Identified Issues**

- Welcome to NEON page: this should be preceded by a message thanking the participant for completing the enrollment process.
- Useful information page: this page includes the text, “It is normal to have a range of strong emotional responses to stories.” This made the stories sound scary and made the participant feel abnormal if they did not have a strong emotional response.
- Initial information page (collects information about the participant for use in the recommender system and allows proactive narrative blocking): more explanation was needed about what this was for. It was not clear what role information would have in matching people to stories. It was difficult to understand the difference between this and the demographics form. Some participants felt they had already provided this information. The titles of content warnings were considered to be very blunt and might cause distress. To get to the Initial Information page, participants have to navigate a substantial number of prior pages without seeing any recovery narratives, some showing warnings about harm. This might cause them to doubt the value of recovery narratives.
- First story page (shows an initial recovery story selected to have no content warnings): it felt quite abrupt to suddenly encounter this story after so many pages of information and data entry. More information was needed about the narrator—there was no explanation as to why this particular story had been chosen.
- Narrative feedback questions: six participants found it difficult to separate the meaning of the two narrative feedback questions on the connection to the narrator or to the story. Several participants did not understand what the purpose of the narrative feedback questions was.
- “Get me out of here” button: in total, 15 out of 25 participants explicitly indicated that they liked the inclusion of a button with the named “Get me out of here”; 8 felt that the name was too dramatic and that a more neutral name such as “Quick exit” would be better.
- “I’m upset” page: a third option was missing—how to access informal peer support for someone who wants to talk to others but does not want to contact a formal service. Give information about the local crisis team.
- Content warnings: in total, 15 participants felt that these were a good idea to include, but 1 participant suggested all stories should have content warnings and 2 participants suggested that the current content warnings do not sufficiently capture eating disorders.

Regarding the acceptability of web-based voucher payments for trial participation, 15 participants indicated that this was acceptable, one indicated that web-based vouchers restricted choice, and one mistakenly thought that vouchers had to be spent on the web (when in fact web-based vouchers can be redeemed in a range of shops). In relation to usage prompts, answers included weekly reminders to check in, notifications of new stories, messages sent if the user had not logged in for a while, and messages thanking them for using the system. Messages could be sent by email or SMS text messages.

Feedback in follow-up interviews about using the NEON Intervention was broadly positive, and some participants indicated that access to the interface had helped them with their mental health problems. Some participants wanted direct access to what they described as *inspirational* stories to help lift their mood when they were feeling low. A few participants lost their log-in details, which was a sufficient barrier that they stopped using the system despite the availability of a password reset option. Some reported enjoying access to stories but failing to find someone like them, indicating a need for more diversity in the NEON Collection.

#### Study Objective 4 (Narrative Feedback) and Study Objective 6 (NEON Intervention Usage)

Participants accessed 330 narratives, of which 253 (76.6%) were selected by the participants and 77 (23.3%) were accessed by the participants asking for a randomly selected narrative. Narrative feedback was provided for 31.8% (105/330) of narratives accessed. The mean length of usage (ie, days from first to last log-in) was 9.8 (SD 9.4), with a median of 9 (IQR 1-16) and range of 1-28. At least one narrative was accessed by 25 participants in week 1, 6 participants in week 2, 4 participants in week 3, and 2 participants in week 4. The mean number of sessions was 2.7 (SD 1.9), with a median of 2 (IQR 2-3) and range of 1-8. The logged data on narrative access are summarized in Table 4.

**Table 4:** Narrative accesses calculated from logged data about prototype usage by participants (n=25).

Parameter			Weeks 1-4		Week 1		Week 2		Week 3		Week 4
**Total narratives accessed per participant**											
	Mean (SD)	13.2 (10.7)		10.1 (7.6)		6.6 (3.8)		7.3 (7.6)		4 (1.4)	
	Median (IQR)	9 (5-18)		7 (4-14)		6 (4-10)		5 (2-13)		4 (3-5)	
	Range	3-42		2-30		2-12		1-18		3-5	

The mean number of unique narratives accessed was 9.2 (SD 6.3), with a median of 7 (IQR 4-13), which shows that participants accessed some narratives multiple times. Other key findings include that the function to select a random narrative was used, that usage reduces over the 4-week period, that no participant accessed all available narratives, and that narrative feedback was only provided for a third of the narratives accessed.

### Modifications Made Following Research Study 2

Eight modifications to the trial procedures were implemented in light of the feedback collected through research study 2:

1. To reduce the length of the participant information sheet, some items were restructured to present summary text only, with the option to expand to complete the text if desired.
2. To allow anonymity, the informed consent form was modified to require yes or no responses rather than initials and to indicate that the person providing consent can use an email address that does not include their name if they wish. These changes are in keeping with the UK Health Research Authority guidance on seeking consent by electronic means [83].
3. To reduce usage barriers arising from losing log-in details, a participant can request that all necessary access details (ie, website address, email log-in, and chosen password) be sent to their email address as soon as they have given consent.
4. Short messages of a maximum of two paragraphs were added to explain the purpose of each trial procedure.
5. To clarify the role of baseline data collection, a message is shown before the presentation of the forms, which states that the information is being collected to support trial evaluation only and will not be used for matching narratives to participants.
6. To reduce participant discomfort and allow the participant to complete web-based forms in private if they wish, the same message also indicates that some questions might be perceived as sensitive.
7. To address concerns about ethnicity, subcategories were added, consistent with ethnicity guidance from the United Kingdom Office for National Statistics [84].
8. To support understanding of the data being collected in relation to trial procedures, a title is provided to describe the information that will be collected (eg, *Information about you* for the trial demographics form) and a subtitle is provided to describe the role of this information in the trial (eg, *This information will help us understand who is taking part in our trials*).

To produce a final version of the NEON Intervention, the following six modifications were implemented based on the findings presented in this paper. These modifications were included in the NEON Intervention version deployed in the NEON trials [27].

1. Response rates to the narrative feedback questions were lower than anticipated. To reduce burden and hence potentially increase completion of narrative feedback data, the two questions on connection described in research study 1 were made optional and the interface was adjusted accordingly. The rating scale for all feedback was simplified to a 4-point scale, for example, hope was changed to −1 (less hopeful than before), 0 (no change), 1 (a bit more hopeful), and 2 (much more hopeful).
2. To reflect the NEON Impact Model, two optional narrative feedback questions on learning and empathy were added.
3. For participants who wanted support without involving formal mental health services, the “I’m upset” page was extended with information about online peer support services. Services were selected by the research team based on features provided to support user safety, such as moderation of discussions and reporting mechanisms in the event of receiving abusive or inappropriate messages.
4. To address concerns about the blunt nature of the content warning systems and to clarify which content warning related to eating disorders, the titles of content warnings were updated. Final content warnings comprised the following: abuse or sexual violence; loss of life or endangerment to life; self-harm including eating disorders; violence or aggression; and injustice, prejudice, and discrimination.
5. To address the negative perceptions of some participants that the NEON Intervention looked like a UK NHS website, a study logo was produced using a color scheme that is distinctively different from that employed by the UK NHS and was integrated into the NEON Intervention. The color scheme adopted by the NEON Intervention was updated to match the logo.
6. To improve accessibility, the “Welcome to NEON” and “Useful information” pages were redrafted in collaboration with the NEON LEAP.

## Discussion

### Principal Findings

The NEON Impact Model developed through knowledge integration activity 1 guided the development of the NEON Intervention, including through informing the selection of narrative feedback questions and through the adoption of a mission to build a large, heterogeneous collection, given that this might increase opportunities for comparison, learning, and empathy for a diverse user base. The Impact Model is relevant to interventions that integrate recovery narratives. It might also guide the selection of recovery narratives by clinicians who integrate these into their practice, for example, by structuring discussions with clients as to which narratives might have the most positive impact.

The curation principles developed through knowledge integration activity 2 are grounded in the practical experience of building a substantial preliminary collection and reporting them supports the transparent management of a collection. This study demonstrates that the VOICES typology can be used as a guideline for collection reporting [24]. These principles can be adopted by other collections.

The experimental evaluation of preliminary narrative feedback questions presented in research study 1 provides evidence that these questions can be used as a mechanism to collect feedback on the impact of narratives, and findings on response rates collected in research study 2 have allowed their design to be refined by categorizing questions as optional or mandatory. The final set of questions may be used in other interventions that require narrative feedback. Research study 1 identified a small number of narratives that were hope-promoting for all in the sample but mostly confirmed our prior findings that narratives are not universally hope-promoting [36]. The study also identified substantial participant variability in response to most narratives, indicating the need to tailor narratives to the needs of participants.

The formal evaluation of a prototype of the NEON Intervention in research study 2 provides evidence that it is feasible to use this intervention in a clinical trial and has allowed for the refinement of intervention and trial procedures. Knowledge about the acceptability of web-based trial procedures designed for the NEON trials can inform the design of web-based trial procedures for other web-based interventions, particularly in relation to the selection of measures where we found evidence that measures designed for delivery on paper were associated with distress for some participants. Safety measures developed for the NEON Intervention in knowledge integration activity 3 might also be more generally applicable to other web-based interventions, particularly those presenting challenging materials.

The immediate clinical relevance of these findings is in informing the NEON randomized controlled trials [27]. The NEON Intervention has been finalized and is now (2020-2022) being evaluated in 3 randomized controlled trials that run in parallel and share the same digital infrastructure. Each trial is designed for participants who may or may not use mental health services and who may or may not choose to receive support from mental health workers in using the intervention.

The long-term generalizability of the findings includes the extension of the NEON Intervention from a mental health focus to (1) other clinical populations, including chronic disorders and palliative and end-of-life care, (2) other marginalized communities beyond health care who may benefit from access to narratives from their community, and (3) other languages and cultures. For any extension, the content of the NEON Collection will need to be widened, and such a program is currently underway to develop a multilanguage repository of Indian mental health recovery narratives, called NEON Collection India.

### Strengths and Limitations

The strengths of this study are the mixed methods design to systematically address design issues, the use of multiple groups of participants with diverse mental health problems and experiences of service use, the consistent involvement of people with lived experience of mental health problems at every stage (including the research team and the NEON LEAP), and the use of web-based prototyping to bring concepts to life to obtain ecologically valid feedback.

Limitations of this study include the use of a single regional site for recruitment, the absence of a structured design approach such as the Double Diamond methodology of the Design Council, and the absence of testing of the final version of the trial procedures and NEON Intervention. This last limitation is being addressed through an internal pilot in the NEON Trial.

### Comparison With Prior Work

Given that sharing a recovery narrative is a core component of the work of peer specialists [14], the NEON Impact Model might be compared with models created to describe the impact of peer workers. Gillard et al [85] have identified that change comes about through a peer worker (1) building trusting relationships based on shared lived experience, (2) role modeling individual recovery and living well with mental health problems, and (3) engaging service users with mental health services and the community. These are more relational mechanisms than our own mechanism of *reflecting on personal experience*, presumably due to the recorded nature of recovery narratives that are being received. Synthesizing partial models presented in prior NEON studies [1,36,38] has enabled us to develop a more comprehensive understanding of what can be learned from recovery narratives and what might moderate the impact of narratives.

The variation in feedback received against individual narratives, with some participants finding the same narrative hope-promoting and others finding it pessimism-promoting, suggests a need for the tailoring of narrative selection to the needs of individuals and validates the choice of a recommender system. The need to tailor digital interventions to recipients has long been recognized in health research, including in studies on stroke rehabilitation technologies [80]. Alankus et al [86] selected a target technology (rehabilitation gaming) and systematically demonstrated how to select properties of the technology that might be tailored. During the NEON trials, logging data will be collected from the recommender system, and an analysis of these data should reveal effective approaches to tailoring narrative selections to trial participants.

### Conclusions

Recorded mental health recovery narratives can be integrated into web-based interventions, and it is feasible to conduct an evaluation of such interventions in a clinical trial.

## Abbreviations

CSG: Collection Steering Group
HHI: Herth Hope Index
INCRESE: Inventory of Characteristics of Recovery Stories
LEAP: Lived Experience Advisory Panel
NEON: Narrative Experiences Online
NHS: National Health Service
NIHR: National Institute for Health Research
VOICES: Values and motivations, Organization, Inclusion and exclusion, Control and collaboration, Ethics and legal, Safety and well-being
